# FN1 promotes invasion of RCC via YWHAZ-associated signaling with partial involvement of the PI3K-Akt pathway

**DOI:** 10.3389/fonc.2026.1974418

**Published:** 2026-10-14

**Authors:** Chao Zhang, Yu Gao, Tao Yang, Zheng Feng, Chunmei Ma, Lingdong Lv, Feilong Yang, Zhiyong Lv

**Affiliations:** Department of Urology, General Hospital of Ningxia Medical University, Yinchuan, China

**Keywords:** FN1, PI3K-Akt signaling, renal cell carcinoma, tumor metastasis, YWHAZ

## Abstract

**Purpose:**

Clear cell renal cell carcinoma (ccRCC) accounts for more than 75% of renal cell carcinomas and is characterized by high metastatic potential and resistance to therapy. Fibronectin 1 (FN1), a major extracellular matrix glycoprotein, is overexpressed in various cancers and promotes tumor progression; however, its precise molecular mechanisms in RCC remain unclear.

**Methods:**

We analyzed the expression level of FN1 and its clinical relevance in ccRCC using TCGA database. Subsequently, FN1 was knocked down in ACHN cells, followed by functional assays, RNA sequencing, and Western blot validation. Furthermore, we integrated FN1-interacting proteins from the BioGRID and STRING databases and combined this information with differentially expressed genes to identify key downstream effectors of FN1.

**Results:**

FN1 was significantly upregulated in ccRCC and strongly associated with lymph node metastasis, distant metastasis, and poor overall survival. FN1 knockdown markedly suppressed invasion of ACHN cells, induced transcriptomic alterations, and downregulated key oncogenes, including CSF2, SOX9, and LAMC2. Pathway analysis revealed significant inhibition of the PI3K–Akt signaling axis. Integrating FN1-interacting proteins with the DEGs identified YWHAZ—a known regulator of the PI3K–Akt pathway—as a critical downstream effector of FN1, whose expression was markedly reduced upon FN1 knockdown. Rescue experiments further showed that YWHAZ overexpression substantially restored FN1 knockdown-impaired invasion but only modestly and non-significantly affected Akt phosphorylation.

**Conclusion:**

FN1 promotes invasion in RCC cells at least in part through YWHAZ-dependent mechanisms, with partial involvement of PI3K-Akt signaling and additional pathways likely contributing. These findings suggest that the FN1/YWHAZ axis may serve as a candidate prognostic marker and a potential target for future preclinical investigation in RCC.

## 1 Introduction

Renal cell carcinoma (RCC) is among the most prevalent cancers, with metastasis being the leading cause of its high mortality rate (1). By 2025, renal cell carcinoma (RCC) is projected to rank as the sixth most frequently diagnosed cancer among males, with a notably elevated risk of mortality (2).

Clear cell renal cell carcinoma (ccRCC) is the predominant histological subtype among renal cell carcinomas, comprising more than 75% of all documented cases. It arises primarily from epithelial cells of the proximal renal tubules and demonstrates significant resistance to standard chemotherapy regimens (3, 4). Despite initial treatment, approximately 30% of patients experience disease progression to locally invasive or systemic metastatic forms. Metastases frequently involve the lungs, skeleton, regional lymph nodes, liver, adrenal glands, and intracranial structures, reflecting organ-specific tropism (5, 6). These metastases exhibit organ-specific biological features, resulting in considerable variability in treatment response and clinical outcomes depending on the site of metastasis. Thus, gaining deeper insights into the intricate molecular mechanisms driving ccRCC invasion and metastasis is essential for facilitating early intervention and developing effective therapeutic approaches to prevent and manage metastatic disease.

The extracellular matrix is a dynamic scaffold essential for cell survival, functioning both as a reservoir of bioactive molecules and as a structurally supportive framework with tissue-specific mechanical properties. It plays a pivotal role in modulating key aspects of cell behavior, including movement, growth, differentiation, adhesion, and resistance to cell death (7). Among its major constituents are two principal glycoproteins: laminin and fibronectin (FN) (8, 9). Fibronectin 1 (FN1) is a key component of the extracellular matrix and plays diverse roles in numerous physiological processes, including embryogenesis, wound healing, hemostasis, cell adhesion, and migration (10–12).

FN1 forms a dimeric glycoprotein through the covalent linkage of two homologous but non-identical polypeptide chains via disulfide bonds at their C-terminal regions. Each subunit contains repeated functional modules designated FNI, FNII, and FNIII, which harbor binding sites for various extracellular ligands, including integrins, collagen, and heparin (13). Accumulating evidence indicates that FN1 is frequently overexpressed in human cancers and promotes tumorigenesis, metastasis, and drug resistance (14, 15). Notably, multiple studies have demonstrated that FN1 overexpression in RCC correlates with more aggressive disease features—including advanced tumor stage, high-grade histology, and reduced overall survival (16, 17). Nevertheless, the precise molecular mechanisms underlying FN1’s functional role in RCC remain incompletely understood.

As a key extracellular matrix glycoprotein, FN1 engages diverse molecular partners—including integrins, growth factors, collagens, and proteoglycans—via its modular domains to orchestrate critical cellular functions such as adhesion, motility, intracellular signaling, and extracellular matrix reorganization. For instance, FN1 has been demonstrated to enhance the growth and invasive spread of thyroid carcinoma cells through activation of the NF-κB signaling pathway (18). Moreover, through integrin-mediated activation of the PI3K–Akt signaling pathway, FN1 drives essential cellular processes, including cell survival and proliferation, while suppressing apoptosis (19). Dysregulation of FN1-mediated PI3K–Akt signaling is frequently observed across multiple malignancies. Elevated FN1 expression enhances AKT phosphorylation, thereby activating downstream proliferative and pro-survival programs that ultimately fuel tumor progression and therapeutic resistance (20–22). The PI3K–Akt signaling pathway serves as a central regulator that integrates extracellular cues to coordinate essential cellular processes—including growth, metabolic adaptation, evasion of cell death, and motility. This pathway, when dysregulated, plays a central role in oncogenesis and malignant progression in a broad spectrum of cancers (23–25). In RCC, aberrant activation of the PI3K–Akt signaling axis is strongly associated with more aggressive disease behavior and worse clinical outcomes (26, 27). However, it remains uncertain whether FN1 promotes oncogenic processes in RCC by modulating this signaling pathway.

In this study, analysis of The Cancer Genome Atlas (TCGA) dataset demonstrated that FN1 is markedly overexpressed in ccRCC and correlates with tumor metastasis and adverse clinical outcomes. Subsequently, we knocked down FN1 in ACHN cells and employed a combination of functional assays, transcriptome sequencing, integrative protein–protein interaction network analysis, and pathway validation to systematically dissect its underlying molecular mechanisms. Our findings show that silencing FN1 significantly inhibits RCC cell invasion, attenuates PI3K–Akt/mTORC1 signaling activity, and decreases expression of the key downstream effector YWHAZ. Collectively, this study reveals a potential FN1-driven mechanism that promotes RCC progression via YWHAZ-dependent signaling, with partial involvement of PI3K-Akt activation. Rescue experiments further demonstrated that YWHAZ overexpression substantially restored FN1 knockdown-impaired invasion, while its effect on Akt phosphorylation was only modest and non-significant. This dissociation suggests that YWHAZ may promote invasion through both PI3K-Akt-dependent and PI3K-Akt-independent mechanisms, highlighting the complexity of FN1 signaling networks. These findings highlight its potential as a candidate biomarker and a target for future investigation.

## 2 Materials and methods

### 2.1 Cell culture and transfections

ACHN cells (ZQ0340, Zhongqiao Xinzhou Biotechnology Co., Ltd., China) were cultured in complete medium containing 10% fetal bovine serum (FBS), 100 µg/mL streptomycin, and 100 U/mL penicillin at 37 °C in a 5% CO_2_ humidified incubator. ACHN is a widely used human renal cell carcinoma cell line that has been employed in numerous studies of RCC invasion, metastasis, and PI3K-Akt signaling (28, 29). Although ACHN was originally derived from a patient with papillary RCC, it shares key molecular and phenotypic features with ccRCC—including PI3K-Akt pathway activation, invasive capacity, and responsiveness to FN1 modulation—making it a suitable model for investigating the FN1/YWHAZ axis in RCC. Cell-line authentication was performed by STR profiling, and mycoplasma testing was routinely conducted.

To knock down FN1 expression, three independent siRNAs targeting FN1 (si-FN1-1, si-FN1-2, and si-FN1-3) and a non-targeting control (si-NC) were purchased from Gemma (Suzhou, China). Sequences are listed in **Supplementary Table S1**. Based on consistent and efficient knockdown across both mRNA and protein levels, si-FN1–2 was selected for all subsequent experiments, including RNA sequencing, invasion assays, pathway analysis, and rescue studies. Transfection was carried out using Lipofectamine (Invitrogen, Carlsbad, CA, USA) according to the manufacturer’s protocol. For YWHAZ overexpression, a pcDNA3.1-YWHAZ plasmid (with FLAG tag) was constructed and transfected using the same Lipofectamine protocol. The FLAG antibody (Proteintech, 66008-3-IG) was used to confirm exogenous YWHAZ expression. Cell lysates were harvested 48 hours post-transfection for evaluation of FN1 mRNA and protein levels by RT-qPCR and Western blotting.

### 2.2 Assessment of gene expression

FN1 knockdown efficiency was assessed by RT-qPCR with GAPDH as the reference gene. cDNA was synthesized from total RNA using a Vazyme kit (R323-01) on a Bio-Rad T100 thermocycler and amplified on an ABI QuantStudio 5 system with Hieff SYBR Green Master Mix (YEASEN). Primer sequences are listed in **Supplementary Table S1**. Relative expression was determined by the 2^- ΔΔCT^ method (30) and analyzed by two-way ANOVA in GraphPad Prism 8.0 (San Diego, CA).

### 2.3 Western blot

ACHN cells were lysed on ice in RIPA buffer (PR20001, Proteintech) with protease inhibitors (4693116001, Sigma), followed by 30 min incubation at 4 °C. Lysates were mixed with loading buffer (P1040, Solarbio), boiled for 10 min, and separated on 10% SDS-PAGE gels. Proteins were transferred to 0.45 μm PVDF membranes (ISEQ00010, Millipore), blocked for 1 h at RT, and probed overnight at 4 °C with primary antibodies against FN1 (15613-1-AP, 1:1,000, Proteintech), FLAG (66008-3-IG, 1:1,000, Proteintech; for detection of exogenous FLAG-tagged YWHAZ), p-AKT (4060T, 1:1,000, CST), AKT (10176-2-AP, 1:1,000, Proteintech), GAPDH (60004-1-IG, 1:5,000, Proteintech), and ACTIN (,20536-1-AP 1:5,000, Proteintech). Membranes were then incubated with HRP-conjugated anti-rabbit (SA00001-2, 1:10,000, Proteintech) or anti-mouse (SA00001-1, 1:10,000, Proteintech) IgG for 45 min at RT. Signals were visualized using ECL reagent (P0018FM, Beyotime). Uncropped original Western blot images are provided in the **Supplementary Figures**.

### 2.4 ELISA for secreted FN1

Secreted FN1 in cell culture supernatants was measured using a Human FN1 ELISA Kit (KE00039, Proteintech) according to the manufacturer’s instructions. Briefly, ACHN cells were transfected with si-NC or si-FN1–2 for 48 h, and culture supernatants were collected and centrifuged at 1,000 × g for 10 min to remove debris. Standards and samples were added to pre-coated wells and incubated for 2 h at 37 °C. After washing, detection antibody was added and incubated for 1 h at 37 °C, followed by HRP-conjugated secondary antibody for 30 min. TMB substrate was added, and the reaction was stopped with sulfuric acid. Absorbance was measured at 450 nm using a microplate reader (Multiskan FC, Thermo Fisher Scientific). FN1 concentrations were calculated from a standard curve and normalized to total protein content or cell number. All experiments were performed in triplicate (n = 3 biological replicates).

### 2.5 Invasion assays

Cell invasion was evaluated using Matrigel-coated Transwell inserts (356234, BD Biosciences). 6 × 10^4^ cells in serum-free medium were seeded into the upper chamber, with 600 μL of 10% FBS medium below. After 24 h, membranes were fixed (4% PFA, Beyotime), stained with 0.1% crystal violet (C0121, Beyotime), and imaged at 200× (MF52-N, Mshot) for quantification.

### 2.6 Cell proliferation assay

Cell proliferation was monitored by CCK-8 assay (Yeasen). At 0, 24, 48, and 72 h post-transfection, cells seeded in 24-well plates were incubated with 10 μL CCK-8 reagent (HY-K0301, MedChemExpress) for 3 h at 37 °C/5% CO_2_. Absorbance at 450 nm was measured using a BioTek ELX800 plate reader.

### 2.7 Wound-healing assay

ACHN cells were cultured to 90% confluence, serum-starved for 4 h, and scratched with a sterile 200 μL pipette tip. After PBS washing, cells were incubated in basal medium containing 10% FBS (10091148, Gibco, USA), 100 μg/mL streptomycin, and 100 U/mL penicillin (SV30010, Hyclone, USA) at 37 °C. Wound images were captured at 0 and 21 h at three random fields per well using an inverted microscope (MF52-N, Mshot, China; ×100 magnification). Wound areas were quantified with ImageJ, and the mobility rate was calculated as: (0 h area − 21 h area)/0 h area × 100%.

### 2.8 RNA extraction and sequencing

Genomic DNA was removed from total RNA using RQ1 DNase (Promega). RNA purity (A260/A280), concentration, and integrity (agarose gel) were evaluated. 1.1 μg of RNA was used for mRNA enrichment (VAHTS mRNA Capture Beads 2.0, Vazyme) and strand-specific library construction (VAHTS V8 Kit, Vazyme; NM344 primers), per manufacturer’s protocol. Sequencing was conducted on the DNBSEQ-T7 platform (150 nt paired-end).

### 2.9 RNA-seq raw data clean and alignment

Low-quality reads containing >2 ambiguous bases were removed, and adaptor sequences along with low-complexity regions were trimmed using FASTX-Toolkit (v0.0.13). FASTX-Toolkit was selected because it provides a lightweight, modular suite for preprocessing high-throughput sequencing data (including quality filtering, adaptor trimming, and length filtering), is widely used in RNA-seq pipelines, and is compatible with the DNBSEQ-T7 platform. After adaptor trimming, a minimum length threshold of 16 bp was applied to discard very short residual fragments. This threshold was chosen because fragments shorter than 16 bp are unlikely to align uniquely to the human reference genome (GRCh38) and may increase the risk of spurious mapping. For 150 nt paired-end reads, the majority of reads retained after quality filtering were substantially longer than 16 bp, and this threshold therefore removed only short, low-complexity residual fragments without affecting the overall data quality. Quality-filtered reads were mapped to the human reference genome (GRCh38) using HISAT2 (31) (maximum 4 mismatches), and uniquely aligned reads were quantified at the gene level. Raw integer read counts were used for differential-expression analysis, while transcripts per million (TPM) values were calculated only for visualization (32).

### 2.10 Differentially expressed genes analysis

Differential expression analysis was performed using DESeq2 (Bioconductor, R) (33) on the raw integer read-count matrix. Genes were deemed significantly altered if adjusted FDR < 0.05 and |log_2_FC| ≥ 1 (≥2-fold change). TPM values were used only for PCA and heatmap visualization and were not used as input for DESeq2.

### 2.11 Functional enrichment analysis

GO and KEGG enrichment analyses of DEGs were conducted using KOBAS 2.0 (34–36), with significance assessed by the hypergeometric test and FDR adjusted via Benjamini-Hochberg correction.

### 2.12 Statistical analysis

Data visualization was carried out in R (v4.2.3) using RStudio, with values presented as mean ± SEM. All experiments were performed with at least three independent biological replicates. Comparisons between two groups were analyzed using unpaired Student’s t-test. For comparisons involving three or more groups (e.g., rescue experiments with four groups: NC, si-FN1, OE-YWHAZ, and si-FN1+OE-YWHAZ), one-way ANOVA followed by Tukey’s multiple comparisons test was used. For qPCR analyses involving multiple siRNAs and target genes, two-way ANOVA followed by Tukey’s multiple comparisons test was used, with siRNA treatment and target gene as the two factors. Normality was assessed using the Shapiro-Wilk test, and equal variance was assessed using Levene’s test. Exact P values are reported where possible. A P value < 0.05 was considered statistically significant. Statistical analyses were performed using GraphPad Prism 8.0 (San Diego, CA).

### 2.13 Gene set enrichment analysis

GSEA was performed using clusterProfiler (v4.6.2) (37), with genes ranked by log_2_ fold-change from si_FN1 vs NC comparisons. The ranked list was analyzed against the Hallmark gene sets to detect enriched pathways.

### 2.14 Public database data download and analysis

This study leveraged the TCGA ccRCC dataset. RNA-seq–derived gene expression data from 542 tumor and 72 adjacent normal samples, along with matched clinical information, were obtained via the Genomic Data Commons (GDC) Data Portal (https://portal.gdc.cancer.gov/). Pearson correlation analysis was performed to assess the relationship between FN1 and YWHAZ expression in the TCGA ccRCC cohort. For combined prognostic analysis, a combined FN1+YWHAZ risk score was constructed using multivariable Cox regression coefficients. For all survival analyses (including FN1, YWHAZ, and the combined risk score), patients with incomplete clinical or survival data were first excluded. The optimal cutoff values were determined using maximally selected rank statistics (the surv_cutpoint function in the survminer package) to stratify patients into high- and low-expression (or high- and low-risk) groups. Notably, this method inherently produces unequal group sizes. Kaplan-Meier survival curves were compared using the log-rank test, and hazard ratios (HR) with 95% confidence intervals (CI) were calculated using univariate Cox regression. All survival analyses were performed using R software (v4.2.3) with the survival and survminer packages.

A protein-protein interaction (PPI) network centered on FN1 was built by combining data from STRING (v12.0) and BioGRID (v5.0.251), both restricted to Homo sapiens. For STRING, all seven active interaction sources were enabled with a minimum interaction score of 0.400 (medium confidence) and a maximum of 10 interactors in the first shell. For BioGRID, all manually curated experimental interactions (physical and genetic) were included without a confidence threshold. Since STRING includes functional and predicted associations in addition to physical interactions, and BioGRID includes both physical and genetic interactions, the resulting network should be considered a hypothesis-generation strategy rather than evidence of direct physical interactions.

## 3 Results

### 3.1 FN1 is upregulated in ccRCC and associated with poor prognosis

We analyzed the TCGA ccRCC cohort—comprising 542 tumor and 72 adjacent non-tumor renal samples—and found that FN1 expression was significantly elevated in tumors compared to normal tissues (**Figure 1A**). Further stratified analyses revealed that tumors exhibiting distant metastasis or lymph node involvement displayed significantly elevated FN1 levels (**Figures 1B, C**), suggesting a robust association between FN1 expression and both tumor aggressiveness and metastatic potential. Patients with elevated FN1 expression demonstrated significantly reduced overall survival relative to those with low expression (**Figure 1D**), suggesting FN1’s potential as a survival-associated marker for adverse outcomes. Supporting these findings, publicly available immunohistochemical images from The Human Protein Atlas (HPA) were qualitatively consistent with increased FN1 protein levels in RCC tissue samples (**Figure 1E**). However, these representative images do not constitute quantitative validation in an independent patient cohort. Together, these findings demonstrate that FN1 is markedly overexpressed in ccRCC at the transcriptomic level and correlates strongly with metastatic progression and adverse patient outcomes.

**Figure 1:**
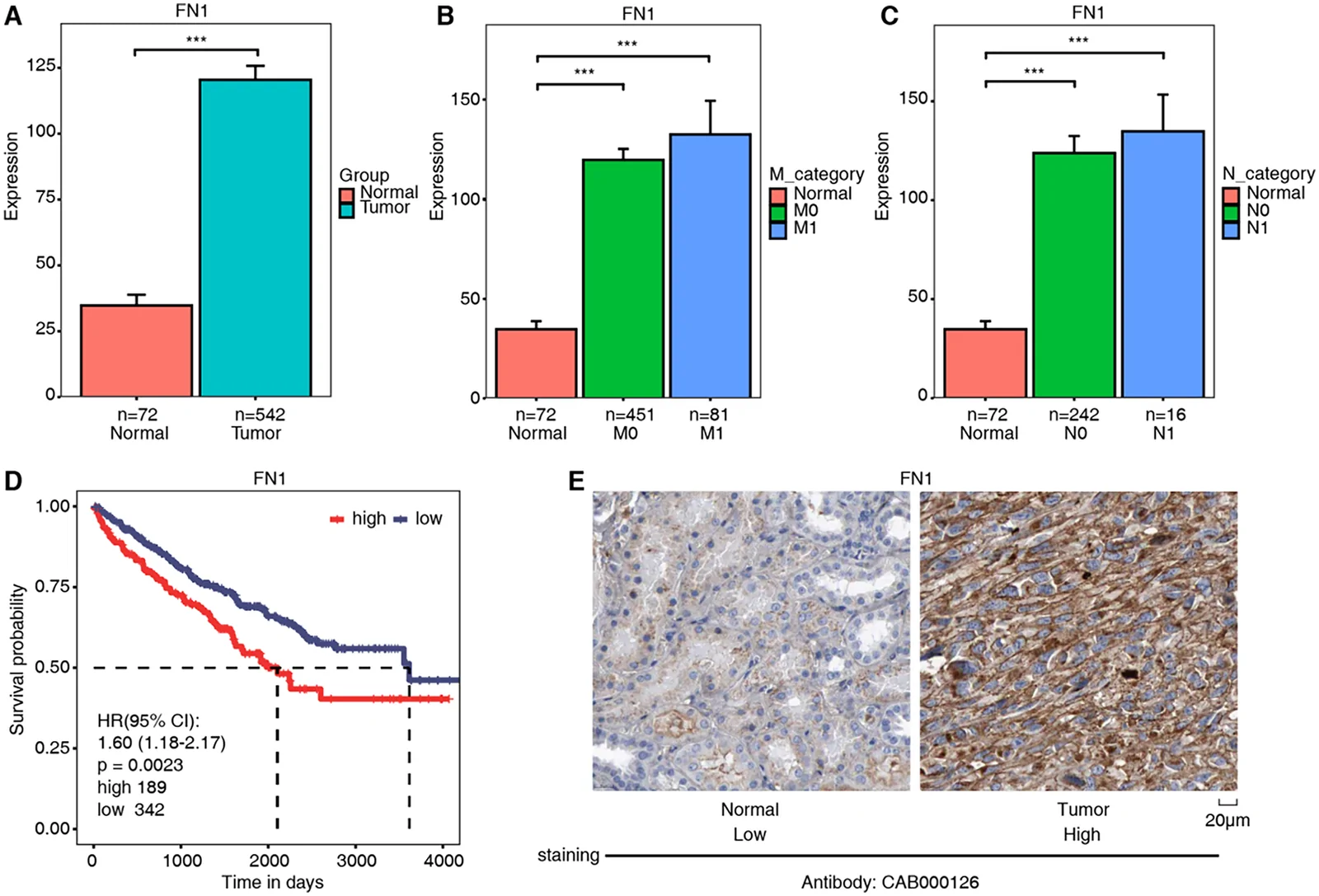
Expression profile and clinical significance of FN1 in RCC. **(A)** FN1 expression in RCC tumors (n = 542) versus adjacent normal kidney tissues (n = 72), analyzed using TCGA data. Data are presented as mean ± SEM. Statistical significance was determined by unpaired Student’s t-test (P < 0.0001). ***P < 0.001. **(B)** FN1 expression stratified by distant metastasis status (M0, n = 451; M1, n = 81; Normal, n = 72) from TCGA. Data are mean ± SEM. One-way ANOVA followed by Tukey’s multiple comparisons test: M0 vs. Normal, P < 0.0001; M1 vs. Normal, P < 0.0001. ***P < 0.001. **(C)** FN1 expression according to lymph node involvement (N0, n = 242; N1, n = 16; Normal, n = 72) from TCGA. Data are mean ± SEM. One-way ANOVA followed by Tukey’s multiple comparisons test: N0 vs. Normal, P < 0.0001; N1 vs. Normal, P < 0.0001. ***P < 0.001. **(D)** Kaplan–Meier survival analysis showing the association between FN1 expression and overall survival in RCC patients. Log-rank test: HR = 1.60 (95% CI: 1.18, 2.17), P = 0.0023; high (n = 189) vs. low (n = 342). **(E)** Representative immunohistochemical images of FN1 in RCC tissues from The Human Protein Atlas (antibody CAB000126). These images are provided for qualitative illustration only and do not constitute quantitative validation.

### 3.2 Knockdown of FN1 suppresses invasion of ACHN cells

To investigate FN1’s role in RCC, we silenced its expression in ACHN cells using three independent siRNAs (si-FN1-1, si-FN1-2, and si-FN1-3). RT-qPCR and Western blot analyses confirmed marked reductions in both FN1 transcript and protein levels for all three siRNAs (**Figures 2A, B**). si-FN1-2, which showed consistent and efficient knockdown at both mRNA and protein levels, was selected for all subsequent experiments, including RNA sequencing, invasion assays, pathway analysis, and rescue studies. ELISA results showed a marked decrease in secreted FN1 in the culture supernatant after knockdown (**Figure 2C**). In addition, FN1 knockdown significantly attenuated ACHN cell invasion, as evidenced by fewer cells penetrating the Matrigel-coated membrane in the Transwell assay (**Figure 2D**). To elucidate the mechanism underlying the attenuated invasive capacity, we performed CCK-8 and migration assays. FN1 knockdown significantly inhibited the proliferation of ACHN cells at 48 and 72 h post-transfection (**Supplementary Figure S1A**), but had no significant effect on their migratory ability (**Supplementary Figure S1B**). Taken together, these data indicate that FN1 promotes the invasive phenotype of ACHN cells, despite a potential contribution from reduced cell proliferation.

**Figure 2:**
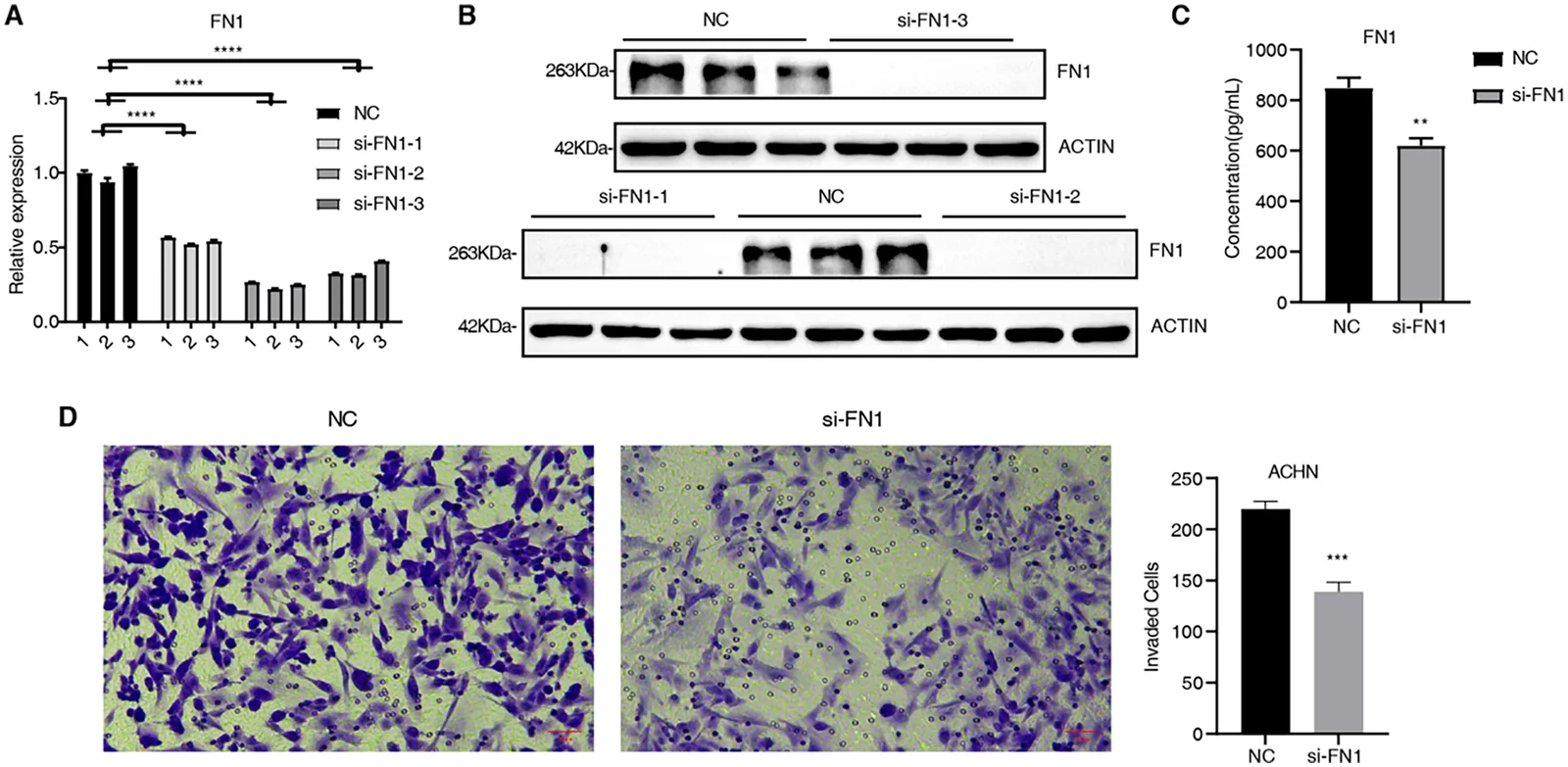
Molecular and functional characterization of FN1 deficiency in ACHN renal carcinoma cells. **(A)** RT-qPCR-based assessment of FN1 transcript levels post-knockdown using three independent siRNAs (si-FN1-1, si-FN1-2, and si-FN1-3). Data are mean ± SEM (n = 3). Two-way ANOVA with Tukey’s test: Row Factor P < 0.01 for all siRNAs; Column Factor P < 0.0001.****P < 0.0001. **(B)** Western blot confirmation of reduced FN1 protein expression using three independent siRNAs (si-FN1-1, si-FN1-2, and si-FN1-3). ACTIN was used as a loading control. **(C)** ELISA quantification of extracellular FN1 in conditioned media. Data are presented as mean ± SEM (n = 3). Statistical significance was determined by unpaired t-test (P = 0.0013). **P < 0.01. **(D)** Invasive behavior evaluated using Matrigel-coated Transwell chambers. Data are presented as mean ± SEM (n = 3). Statistical significance was determined by unpaired t-test (P = 0.0003). ***P < 0.001.

### 3.3 FN1 knockdown alters the transcriptomic profile of ACHN cells

To further investigate the molecular mechanisms of FN1 in RCC, RNA sequencing (RNA-seq) was performed on ACHN cells, comparing a negative control group (NC) with an FN1 knockdown group (si_FN1), each with three biological replicates. RNA-seq quality-control metrics, including library size, mapping rate, replicate-level QC, and dispersion estimates, confirmed high data quality (**Supplementary Tables S2**– **S4**; **Supplementary Figure S2**). Specifically, each sample yielded an average of 86.4 million high-quality paired-end reads … with an average mapping rate of ~97% (**Supplementary Table S2**). Principal component analysis (PCA) based on genome-wide TPM values clearly distinguished the two groups at the transcriptomic level (**Figure 3A**), suggesting that FN1 silencing significantly altered the transcriptional profile of ACHN cells. Knockdown of FN1 induced widespread transcriptional changes, with 103 genes significantly upregulated and 120 downregulated compared to control conditions (**Figure 3B**, **Supplementary Table S5**). Hierarchical clustering of DEGs highlighted distinct expression patterns between the two conditions, with consistent results across biological replicates (**Figure 3C**).

**Figure 3:**
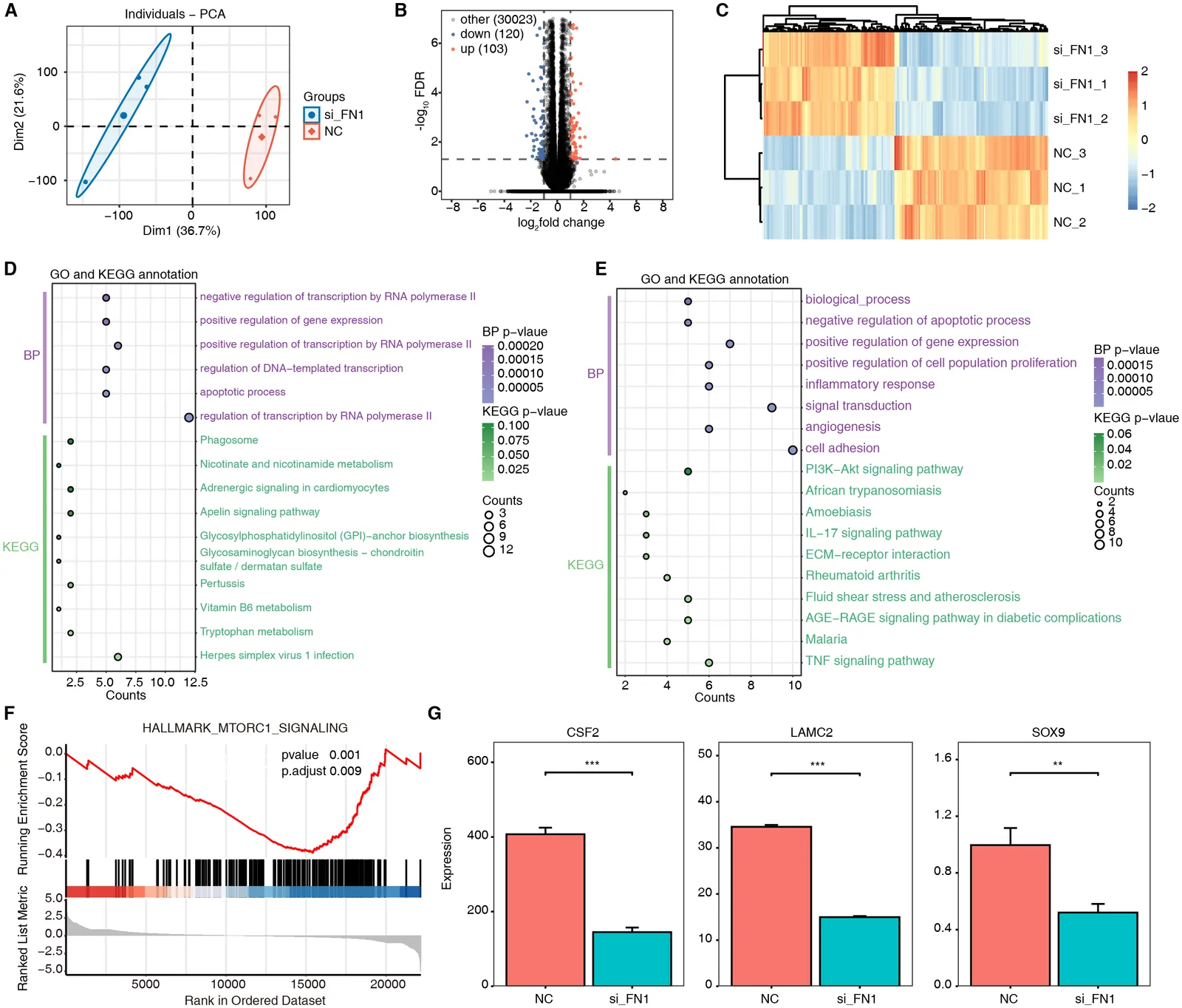
Changes in gene expression profiles of ACHN cells following FN1 knockdown. **(A)** PCA plot based on all expressed genes. **(B)** Volcano plot identifying DEGs in si_FN1 versus si_NC cells. Horizontal dashed line: adjusted P = 0.05; vertical dashed lines: log2FC = ± 1. Genes with adjusted P < 0.05 and |log2FC| ≥ 1 are considered significantly differentially expressed. **(C)** Hierarchical clustering heatmap of DEGs. **(D, E)** Functional enrichment analysis of upregulated and downregulated DEGs via GO and KEGG pathways. **(F)** GSEA highlighting enrichment of the mTORC1 signaling cascade. **(G)** Quantitative expression of key genes CSF2, SOX9, and LAMC2. Data are derived from RNA-seq (n = 3). Differential expression was determined by DESeq2 with FDR-adjusted P values (CSF2: padj = 1 × 10^-88^; SOX9: padj = 0.0048; LAMC2: padj = 1 × 10^-110^). **P < 0.01, ***P < 0.001 (FDR-adjusted).

To better understand the biological functions of DEGs following FN1 knockdown, functional enrichment was performed using GO and KEGG pathway databases. Enrichment of upregulated genes was observed in GO biological processes (GO-BP) terms such as RNA polymerase II-mediated transcription regulation, apoptosis, and gene expression regulation, and in KEGG pathways including adrenergic signaling in cardiomyocytes and phagosome formation (**Figure 3D**). Conversely, downregulated genes were overrepresented in GO-BP—particularly cell adhesion, angiogenesis, inflammatory response, cell proliferation regulation, and apoptotic modulation. KEGG pathway mapping further linked these genes to key signaling cascades, notably TNF, IL-17, extracellular matrix–receptor interaction, and PI3K–Akt pathways (**Figure 3E**).

Moreover, GSEA demonstrated significant suppression of the mTORC1 signaling pathway upon FN1 knockdown (**Figure 3F**). Given that mTORC1 is a key downstream effector of the PI3K–Akt cascade, FN1 is likely to modulate tumor-associated biological processes via the PI3K–Akt/mTORC1 signaling axis. Indeed, accumulating data indicate that the PI3K–Akt pathway drives key hallmarks of cancer, including enhanced proliferation, invasive capacity, cell motility, and resistance to apoptosis (23–25). Notably, the downregulation of invasion-related genes (CSF2, SOX9, LAMC2) upon FN1 knockdown (**Figure 3G**) underscores FN1’s functional importance in promoting renal cell carcinoma malignancy.

### 3.4 YWHAZ-associated signaling YWHAZ-associated signaling is involved in FN1 promotion of invasion in ACHN cells

FN1 is a highly versatile extracellular matrix glycoprotein that binds to numerous proteins and is critically involved in cell adhesion, migration, signal transduction, and tissue remodeling. Considering that mTORC1 functions as a central downstream effector of the PI3K–Akt signaling cascade, we investigated whether FN1 modulates key nodal components of this pathway to affect RCC progression.

Since mTORC1 serves as a central downstream effector of the PI3K–Akt signaling pathway, we investigated whether FN1 modulates key nodal components within this cascade to impact RCC progression. To address this, we combined data from the STRING and BioGRID databases to identify 815 putative FN1-interacting proteins (**Figure 4A**). Subsequent GO and KEGG enrichment analyses of these interactors showed significant enrichment in biological processes such as translation, protein stabilization, RNA splicing, platelet aggregation, and regulation of apoptosis. KEGG pathway analysis indicated that these proteins were mainly involved in pathways such as ribosome biogenesis, spliceosome function, proteoglycans in cancer, complement and coagulation cascades, and focal adhesions (**Figure 4B**). Further intersection analysis between the 815 predicted interacting proteins and DEGs identified after FN1 knockdown revealed 6 genes whose expression was significantly altered upon FN1 knockdown (**Figure 4C**). Notably, among these genes, YWHAZ—a key modulator of the PI3K–Akt signaling pathway—exhibited a marked reduction in mRNA expression upon FN1 knockdown, as validated by qPCR (**Figure 4D**). Consistent with this, FN1 depletion was associated with reduced PI3K-Akt signaling, as evidenced by a significantly lower p-Akt/Akt ratio in knockdown cells compared to negative controls in this experiment (**Figure 4E**). In summary, these results suggest that YWHAZ was identified as a candidate downstream effector associated with the FN1 network, indicating that FN1 may promote RCC progression by maintaining PI3K-Akt signaling activity through YWHAZ-associated mechanisms.

**Figure 4:**
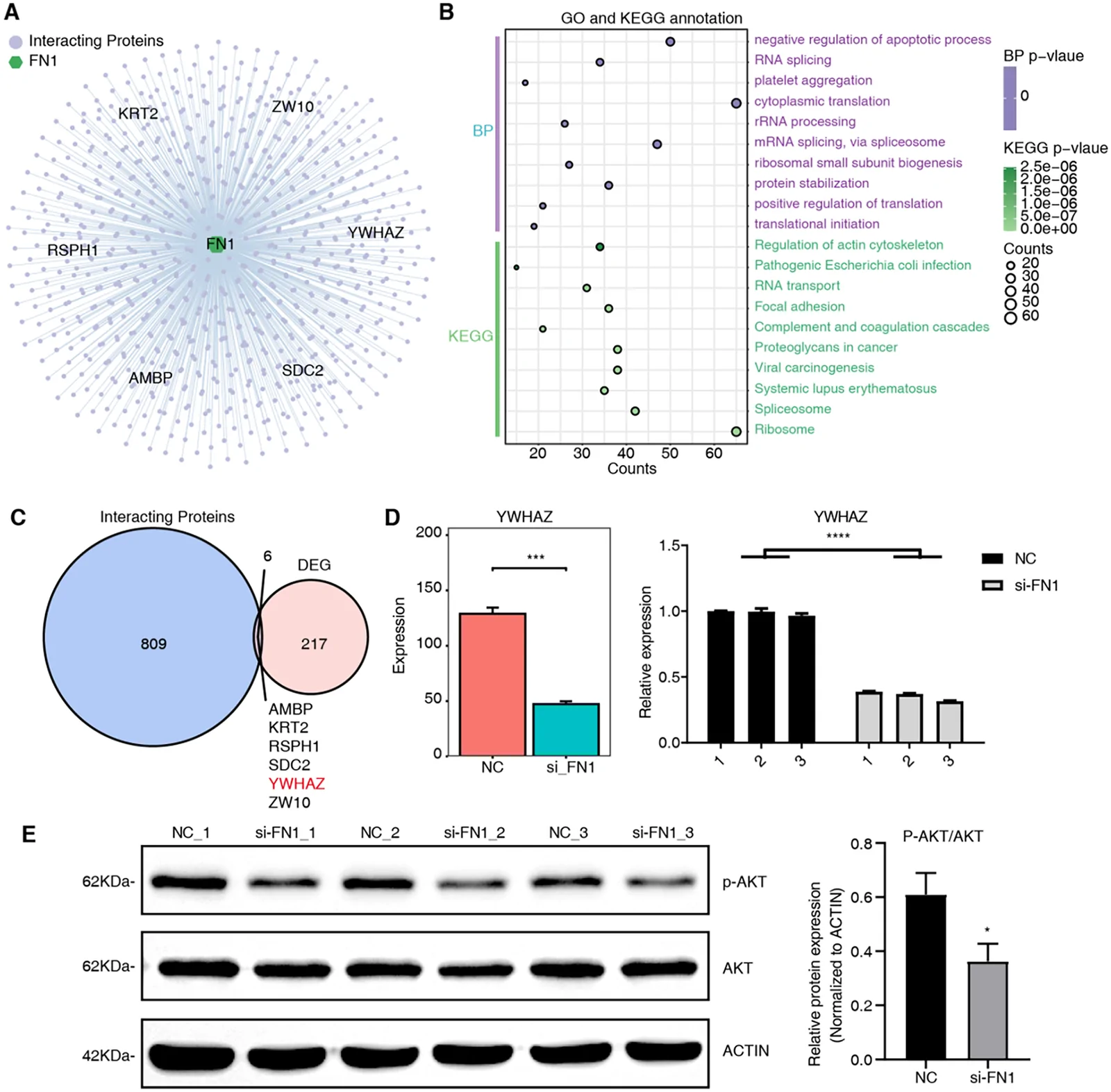
FN1 is associated with PI3K-Akt signaling and YWHAZ as a candidate downstream effector. **(A)** protein interaction network of potential FN1-interacting proteins constructed using data from the STRING and BioGRID databases. **(B)** GO/KEGG enrichment analysis of the predicted FN1-interacting proteins. **(C)** Venn diagram illustrating the overlap between FN1-interacting proteins and DEGs identified upon FN1 knockdown. **(D)** mRNA expression level of YWHAZ following FN1 knockdown, with validation by qPCR. Data are derived from RNA-seq (n = 3 per group). Differential expression was determined by DESeq2 with FDR-adjusted P values (padj = 1 × 10^-85^). ***P < 0.001 (FDR-adjusted). **(E)** Western blot analysis of p-Akt and total Akt protein levels; bar graph shows quantification of the p-Akt/Akt ratio. Data are presented as mean ± SEM (n=3). Statistical significance was determined by unpaired t-test (P = 0.0139, effect size R² = 0.8139). *P < 0.05; ****P < 0.0001.

### 3.5 YWHAZ expression profile and clinical significance in ccRCC, and combined prognostic analysis with FN1

To assess the clinical relevance of YWHAZ in ccRCC, we performed an additional analysis using the ccRCC cohort from TCGA database. YWHAZ expression was significantly higher in ccRCC tissues than in adjacent non-tumorous kidney tissues (**Figure 5A**). Moreover, YWHAZ expression was further elevated in tumors with distant metastasis or lymph node involvement (**Figures 5B, C**), suggesting a strong association between YWHAZ levels and tumor aggressiveness and metastatic potential. High YWHAZ expression correlated with poor prognosis, as reflected by markedly shorter overall survival in the patient cohort (**Figure 5D**).

**Figure 5:**
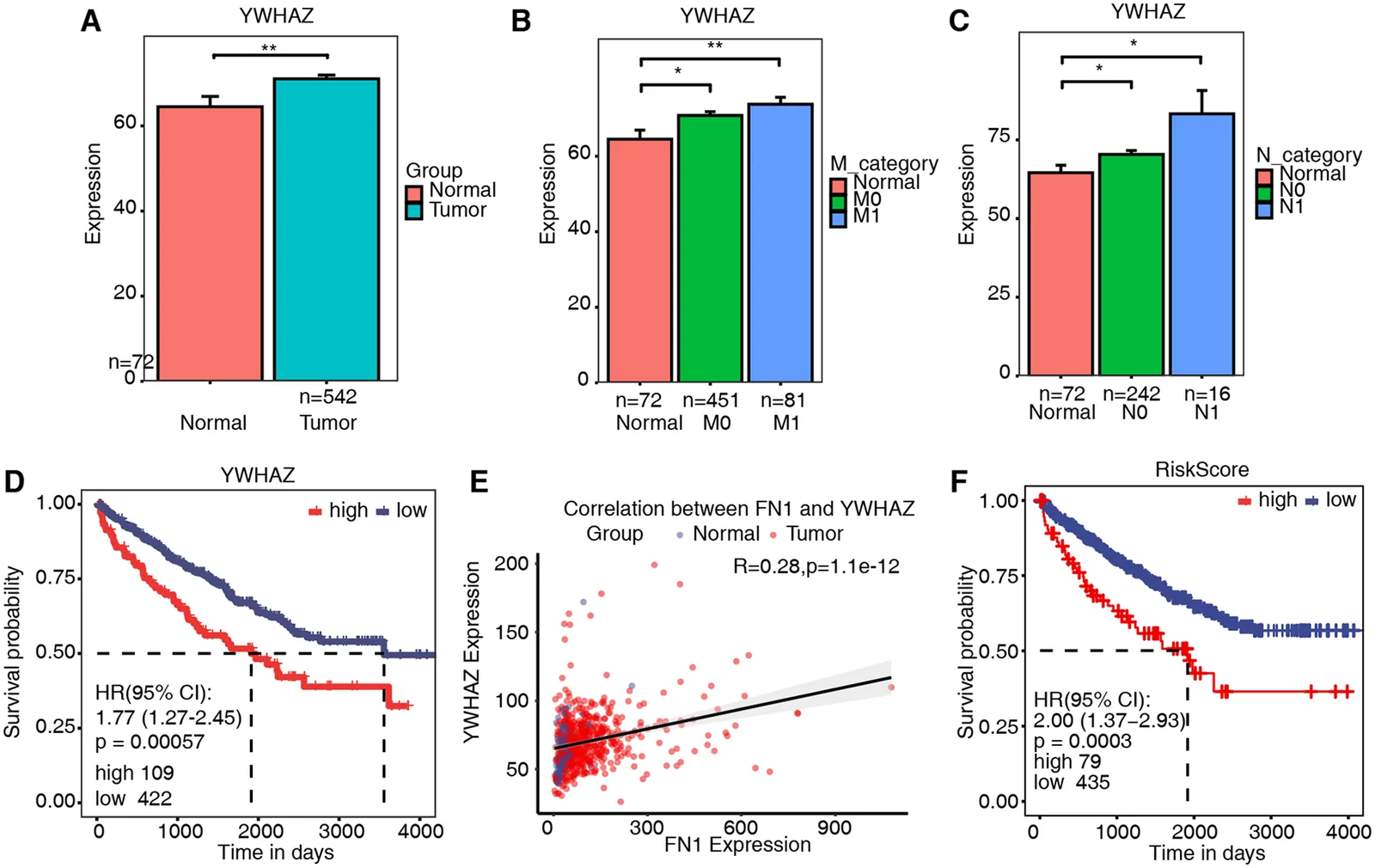
Expression profile and clinical significance of YWHAZ in RCC. **(A)** YWHAZ expression in RCC tumors (n = 542) versus adjacent normal kidney tissues (n = 72), analyzed using TCGA data. Data are presented as mean ± SEM. Statistical significance was determined by unpaired Student’s t-test (P = 0.0026). **P < 0.01. **(B)** YWHAZ expression stratified by distant metastasis status (Normal, n = 72; M0, n = 451; M1, n = 81) from TCGA. Data are mean ± SEM. One-way ANOVA followed by Tukey’s multiple comparisons test: M0 vs. Normal, P = 0.0166; M1 vs. Normal, P = 0.0027. *P < 0.05, **P < 0.01. **(C)** YWHAZ expression according to lymph node involvement (Normal, n = 72; N0, n = 242; N1, n = 16) from TCGA. Data are mean ± SEM. One-way ANOVA followed by Tukey’s multiple comparisons test: N0 vs. Normal, P = 0.033; N1 vs. Normal, P = 0.0267. *P < 0.05. **(D)** Association between YWHAZ expression and overall survival in ccRCC patients. Log-rank test: HR = 1.77 (95% CI: 1.27, 2.45), P = 0.00057; high (n = 109) vs. low (n = 422). **(E)** Correlation between FN1 and YWHAZ mRNA expression in the TCGA RCC cohort (n = 614). Pearson correlation analysis: r = 0.28, P < 0.001. **(F)** Kaplan–Meier curves for overall survival of ccRCC patients stratified by the combined FN1+YWHAZ Risk Score. Log-rank test: HR = 2.00 (95% CI: 1.37, 2.93), P = 0.0003; high-risk (n = 79) vs. low-risk (n = 435).

To evaluate the clinical relevance of the proposed FN1/YWHAZ axis, we examined the correlation between FN1 and YWHAZ expression in the TCGA ccRCC cohort. Pearson correlation analysis revealed a significant positive correlation between FN1 and YWHAZ expression (**Figure 5E**). We next evaluated whether combined FN1/YWHAZ expression provides stronger prognostic information than either marker alone. A combined FN1+YWHAZ risk score was constructed using multivariable Cox regression coefficients. Patients were stratified into high-risk (n = 79) and low-risk (n = 435) groups using the optimal cutoff value via maximally selected rank statistics (surv_cutpoint). Kaplan-Meier survival analysis showed that patients in the high-risk group had significantly shorter overall survival compared to the low-risk group (**Figure 5F**). Notably, the combined risk score showed a higher hazard ratio (HR = 2.00, P = 0.0003) than either FN1 alone (HR = 1.60, P = 0.0023) or YWHAZ alone (HR = 1.77, P = 0.00057), suggesting that the combined FN1/YWHAZ signature may provide improved prognostic stratification compared to either marker individually. Together, these results support a model in which FN1 and YWHAZ cooperate in promoting RCC progression and adverse clinical outcomes.

### 3.6 Effects of YWHAZ overexpression on FN1 knockdown-induced suppression of invasion and PI3K-Akt signaling

To determine whether YWHAZ serves as a critical downstream mediator of FN1 in RCC, rescue experiments were performed by simultaneously knocking down FN1 and overexpressing YWHAZ in ACHN cells. RT-qPCR and Western blot analyses confirmed that FN1 expression was markedly reduced in the siFN1 group, while YWHAZ was significantly upregulated in the oeYWHAZ group. In the combined treatment group (si-FN1+OE-YWHAZ), FN1 remained suppressed whereas YWHAZ expression was successfully restored (**Figures 6A, B**), confirming the effectiveness of the co-manipulation. Transwell invasion assays revealed that FN1 knockdown significantly impaired cell invasion, whereas YWHAZ overexpression significantly enhanced it. Notably, the invasive ability of the combined treatment group was markedly recovered compared with the si-FN1 alone group, reaching levels comparable to those of the control cells (**Figure 6C**), indicating that YWHAZ overexpression effectively compensates for the invasion defect caused by FN1 deficiency. We next examined the activation status of the PI3K-Akt pathway by Western blotting. The results showed that following FN1 knockdown alone, the p-Akt/Akt ratio exhibited a decreasing trend; YWHAZ overexpression alone significantly increased this ratio. In the combined treatment group, the p-Akt/Akt ratio was lower than that in the OE-YWHAZ alone group and slightly higher than that in the si-FN1 alone group (**Figure 6D**).

**Figure 6:**
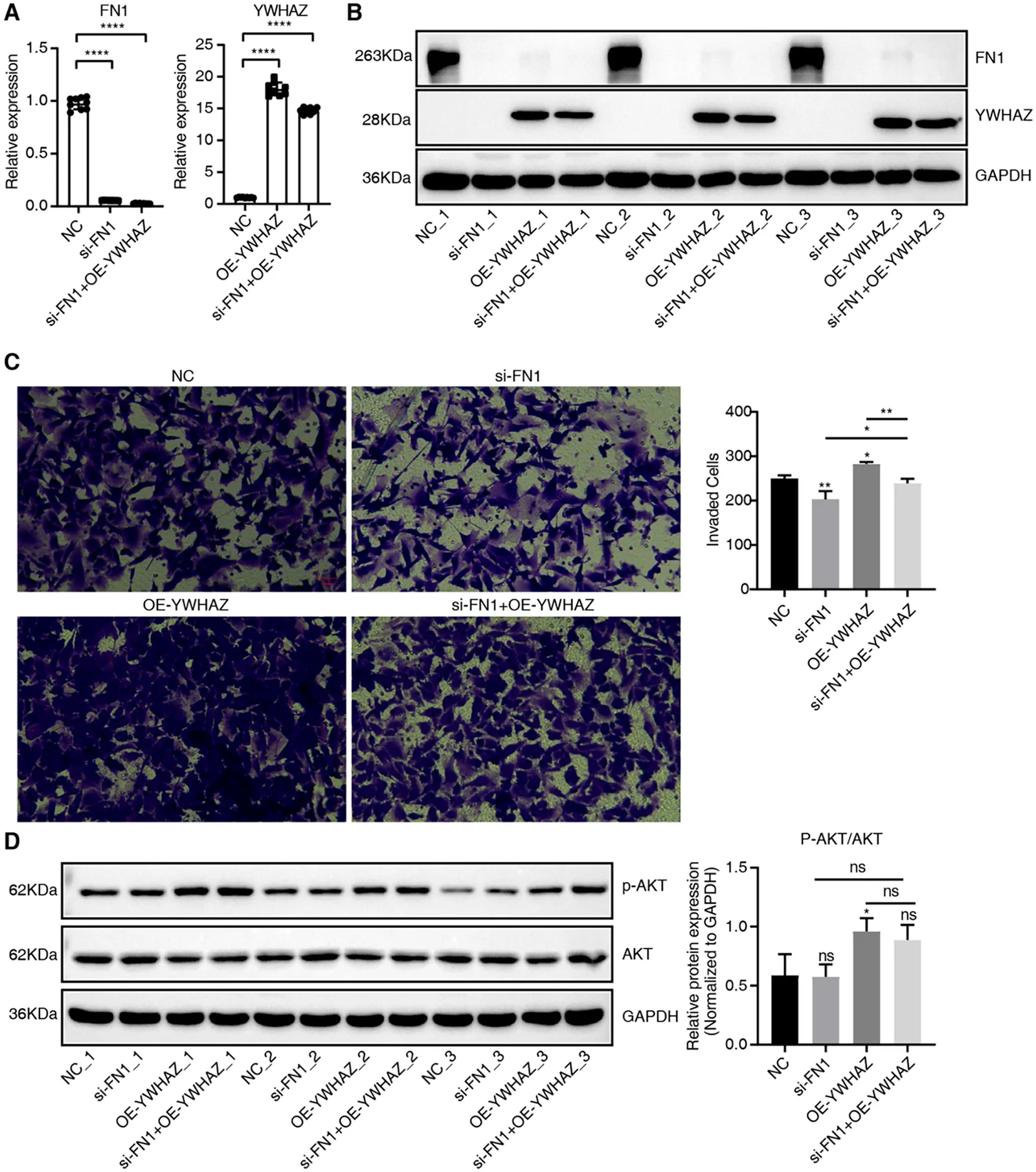
YWHAZ overexpression rescues FN1 knockdown-induced suppression of invasion and PI3K-Akt signaling in ACHN cells. **(A)** RT-qPCR analysis of FN1 and YWHAZ mRNA levels in NC, si-FN1, OE-YWHAZ, and si-FN1+OE-YWHAZ groups. Data are presented as mean ± SEM (n = 3). Statistical significance was determined by one-way ANOVA followed by Tukey’s multiple comparisons test (P<0.0001). ****P<0.0001. **(B)** Western blot validation of FN1 and YWHAZ protein expression. **(C)** Representative images and quantification of Transwell invasion assays. Data are presented as mean ± SEM (n = 3). Statistical significance was determined by one-way ANOVA followed by Tukey’s multiple comparisons test (NC vs. si-FN1, P = 0.0047; NC vs. si-FN1+OE-YWHAZ, P = 0.0328; si-FN1 vs. si-FN1+OE-YWHAZ, P = 0.0202; OE-YWHAZ vs. si-FN1+OE-YWHAZ, P = 0.0073). *P < 0.05, **P < 0.01. **(D)** Representative Western blots and densitometric analysis of p-Akt and total Akt protein levels. Data are presented as mean ± SEM (n = 3). Statistical significance was determined by one-way ANOVA followed by Tukey’s multiple comparisons test. The overall analysis indicated a significant difference among groups (P = 0.0143; effect size Ri = 0.7148). Specific comparisons showed significant upregulation in the OE-YWHAZ group compared to NC (P = 0.0376), while other comparisons were not significant (e.g., NC vs. si-FN1, P = 0.9994; NC vs. si-FN1+OE-YWHAZ, P = 0.0963; si-FN1 vs. si-FN1+OE-YWHAZ, P = 0.0818; OE-YWHAZ vs. si-FN1+OE-YWHAZ, P = 0.9062). *P < 0.05; ns, not significant.

Notably, the significant reduction in the p-Akt/Akt ratio observed in **Figure 4E** (P = 0.0139) was not reproduced as a statistically significant decrease in the si-FN1 group in this independent experiment (P = 0.9994), although a consistent decreasing trend was observed. This discrepancy may reflect biological variability between independent experiments, differences in transfection efficiency, or limited statistical power due to the small sample size (n = 3 per group). Importantly, YWHAZ overexpression did not significantly rescue Akt phosphorylation in FN1-knockdown cells (P = 0.0818), suggesting that the restoration of invasion by YWHAZ may not be primarily mediated through PI3K-Akt activation. Taken together, these results suggest that the regulatory effect of FN1 on Akt phosphorylation may be partially dependent on YWHAZ, but YWHAZ is not the sole mediator. Importantly, the dissociation between phenotypic rescue (substantial restoration of invasion) and signaling rescue (non-significant restoration of Akt phosphorylation) indicates that YWHAZ may restore invasion through mechanisms that are at least partially independent of PI3K-Akt activation. FN1 may also participate in sustaining PI3K-Akt signaling through other parallel pathways, while YWHAZ may promote invasion via PI3K-Akt-independent routes.

## 4 Discussion

RCC ranks as the sixth most frequently diagnosed malignancy among men and exhibits aggressive clinical behavior, with a strong tendency toward metastasis and poor survival outcomes. ccRCC accounts for more than 75% of all cases and exhibits marked resistance to conventional chemotherapy (2–4). Metastasis is the primary driver of poor prognosis in RCC patients, and the heterogeneity across different metastatic sites further complicates therapeutic management (5, 6). FN1 is overexpressed across multiple cancer types and actively modulates key cellular processes—including cell adhesion, migration, and signal transduction. Previous studies have linked elevated FN1 expression in RCC to advanced tumor stage, high histological grade, and poor survival (16, 17); however, the exact molecular pathways through which it exerts its effects have not been fully elucidated. In this study, we found that FN1 is significantly upregulated in RCC and strongly associated with adverse clinical outcomes. FN1 knockdown significantly impaired renal cancer cell invasion, concomitant with attenuation of PI3K–Akt/mTORC1 signaling—an effect likely mediated by downregulation of the key regulator YWHAZ. Collectively, our findings indicate that FN1 may contribute to RCC progression at least in part through YWHAZ-dependent signaling, with partial involvement of PI3K-Akt signaling. These findings reveal a potential mechanistic role of the FN1/YWHAZ axis in promoting invasion in RCC cells and provide a rationale for its application in prognostic assessment and future preclinical investigation.

Prior research has demonstrated that FN1 is upregulated in RCC and strongly correlated with unfavorable pathological characteristics (16, 17). As a central constituent of the extracellular matrix, FN1 not only offers structural scaffolding for tumor cells but also exerts a substantial impact on their behavior through the regulation of gene expression networks (38, 39). In this study, we observed that FN1 knockdown significantly suppressed the invasive ability of ACHN cells—a phenotypic shift that aligns closely with the transcriptome-wide remodeling revealed by RNA-seq. GSEA of downregulated genes revealed significant enrichment in biological processes including cell adhesion, angiogenesis, and regulation of cell population proliferation. Notably, FN1 knockdown led to a significant downregulation of invasion-related genes (CSF2, SOX9, LAMC2), underscoring FN1’s functional importance in promoting the malignant phenotype of ACHN cells (40, 41). SOX9, a key transcriptional regulator, is critically involved in sustaining cancer stemness and driving epithelial–mesenchymal transition (42–44). LAMC2, a member of the laminin family, is frequently overexpressed at the invasive front of aggressive tumors and facilitates cancer cell breaching of the basement membrane (45, 46). The coordinated downregulation of these critical effectors suggests that FN1 may sustain the aggressive malignant phenotype of RCC by preserving levels of these downstream oncogenic molecules.

Earlier studies have indicated that FN1 contributes to malignant progression through potentiation of PI3K–Akt signaling (47). Our study further uncovers a potential mechanistic link by which FN1 may regulate this pathway via YWHAZ-associated signaling. Extracellular matrix proteins typically transduce signals into the cell via integrin receptors and other surface molecules (48), our PPI network analysis and experimental validation suggest that the intracellular adaptor protein YWHAZ may serve as a critical node in this process. As a member of the 14-3–3 protein family, YWHAZ is known to bind phosphorylated serine/threonine motifs on client proteins, thereby protecting key signaling molecules from dephosphorylation or proteasomal degradation and sustaining pro-survival signaling (49, 50). Indeed, prior evidence has demonstrated that YWHAZ promotes tumorigenesis by activating the PI3K–Akt–mTOR cascade (51, 52). Consistent with this, our results show that FN1 knockdown significantly reduces YWHAZ mRNA expression and markedly suppresses Akt phosphorylation as well as downstream mTORC1 pathway activity. These findings suggest that FN1 may contribute to sustaining PI3K-Akt signaling, at least in part by maintaining YWHAZ expression, thereby contributing to the invasive phenotype. Importantly, clinical data from TCGA corroborate the pathological relevance of this mechanism: both FN1 and YWHAZ are significantly overexpressed in ccRCC, and their elevated expression levels correlate strongly with nodal involvement, distant metastasis, and adverse clinical outcomes. Moreover, FN1 and YWHAZ expression were significantly positively correlated in the TCGA ccRCC cohort, and a combined FN1+YWHAZ risk score provided stronger prognostic stratification (HR = 2.00, P = 0.0003) than either FN1 alone (HR = 1.60, P = 0.0023) or YWHAZ alone (HR = 1.77, P = 0.00057). Although the correlation strength was modest, it may reflect the complexity of FN1/YWHAZ regulation, which could involve additional layers of control such as post-transcriptional regulation or protein stability. These findings suggest that the two markers may cooperate in promoting ccRCC progression and that their combined assessment may improve prognostic prediction. Collectively, these results indicate that aberrant activation of the FN1/YWHAZ axis is associated with RCC invasion and reduced survival, underscoring its potential relevance for prognostic assessment and future preclinical investigation.

To directly test whether YWHAZ contributes to the pro-invasive effects of FN1, we performed rescue experiments by overexpressing YWHAZ in FN1-knockdown ACHN cells. Notably, YWHAZ overexpression substantially restored the invasive capacity of FN1-deficient cells, yet it only marginally and non-significantly increased the p-Akt/Akt ratio. This dissociation between phenotypic rescue and signaling restoration is an important finding that suggests YWHAZ may restore invasion through mechanisms that are at least partially independent of PI3K-Akt activation. Several possibilities may explain this observation.

First, FN1 may promote invasion through both YWHAZ-dependent PI3K-Akt signaling and additional YWHAZ-independent mechanisms. For instance, FN1 could activate other downstream kinases (e.g., MAPK/ERK) or transcription factors that converge on invasive programs (53). Second, YWHAZ, as a member of the 14-3–3 protein family, is known to interact with numerous client proteins beyond PI3K-Akt components, including regulators of cytoskeletal dynamics, cell polarity, and epithelial-mesenchymal transition (EMT) (49, 50). For example, YWHAZ has been shown to promote EMT and metastasis through a β-catenin-dependent mechanism in lung cancer (54), and to modulate glycolysis in ovarian cancer (52). Therefore, YWHAZ may regulate cell motility and invasion through PI3K-Akt-independent routes such as Wnt/β-catenin signaling, MAPK/ERK cascades, or cytoskeletal reorganization. Third, the partial and non-significant rescue of Akt phosphorylation may reflect the existence of parallel FN1-driven pathways that sustain PI3K-Akt activity independently of YWHAZ, or may indicate that the PI3K-Akt axis is not the primary downstream effector of YWHAZ in this context.

Nevertheless, the partial trend toward rescue of Akt phosphorylation supports the notion that YWHAZ contributes, at least in part, to FN1-mediated PI3K-Akt signal transduction. However, the substantial restoration of invasion despite non-significant Akt rescue underscores YWHAZ as a key node in FN1-driven invasion, while highlighting that PI3K-Akt activation is not the sole mechanism through which YWHAZ promotes invasion. These findings reinforce the complexity of FN1 signaling networks and highlight the need for further investigation into parallel pathways that cooperate with the FN1/YWHAZ axis to drive RCC aggressiveness. Future studies should include pharmacological or genetic inhibition of PI3K-Akt during YWHAZ rescue to directly test pathway dependency, as well as unbiased approaches such as phosphoproteomics to identify additional YWHAZ effectors.

Collectively, our study provides a plausible mechanistic framework for how FN1 contributes to renal cell carcinoma progression. FN1 not only shapes the tumor microenvironment by providing structural support and modulating cell adhesion and migration, but may also promote invasion by maintaining YWHAZ levels, with partial involvement of PI3K-Akt/mTORC1 signaling. Together, these findings reveal a pro-invasive molecular network associated with the FN1-YWHAZ axis, which may contribute to the aggressive behavior of ccRCC, though not exclusively, as our rescue experiments showed that YWHAZ overexpression substantially restored invasion but only modestly and non-significantly affected Akt phosphorylation. Therefore, the FN1/YWHAZ axis represents a candidate for future preclinical investigation, and further studies using pharmacological inhibitors, additional RCC cell models, and *in vivo* metastasis assays are warranted to validate its therapeutic potential. Our work provides a foundation for future research into survival-associated marker and targeted interventions centered on this signaling axis.

### 4.1 Limitations

This study has several limitations. First, all functional experiments were performed in ACHN cells, which were originally derived from papillary RCC and may not fully recapitulate the molecular heterogeneity of ccRCC. Validation in additional molecularly distinct RCC/ccRCC cell models (e.g., 786-O or Caki-1) is needed to establish generalizability. Second, the RNA-seq analysis was based on three biological replicates per group, which may provide limited statistical power. Third, the FN1-YWHAZ interaction was inferred from database predictions (STRING, BioGRID) and not validated by direct biochemical experiments; therefore, YWHAZ should be considered a candidate downstream effector rather than a confirmed direct binding partner. Fourth, the evidence that YWHAZ-mediated invasion depends on PI3K-Akt activation is limited: the effect of FN1 knockdown on Akt phosphorylation was not entirely consistent across experiments (**Figure 4E**: P = 0.0139; **Figure 6D**: P = 0.9994), and YWHAZ overexpression did not significantly rescue Akt phosphorylation (P = 0.0818). Additional downstream markers (e.g., p-mTOR, p-S6K) were not evaluated, and future studies with larger sample sizes and pharmacological or genetic inhibition of PI3K-Akt during YWHAZ rescue are warranted. Fifth, although three independent FN1-targeting siRNAs were validated, an siRNA-resistant FN1 rescue experiment was not performed, and the possibility of off-target effects cannot be entirely excluded. Sixth, FN1 knockdown significantly inhibited cell proliferation, and the possibility that reduced proliferation partly contributes to the decreased number of invaded cells cannot be entirely excluded. Seventh, the TCGA prognostic analysis was retrospective and based on univariate Cox regression without multivariable adjustment for age, tumor stage, grade, and TNM status; FN1 and YWHAZ should therefore be considered survival-associated markers rather than independent prognostic biomarkers. Eighth, no *in vivo* metastasis model was used, and the association with metastasis was inferred from TCGA clinical data; statements regarding metastasis should be considered hypothesis-generating. Finally, the HPA images were used for qualitative illustration only and do not constitute quantitative validation; future studies should include quantitative IHC or proteomic analysis and independent patient cohorts to confirm the clinical relevance of the FN1/YWHAZ axis.

## 5 Conclusion

FN1 promotes invasion in RCC cells at least in part through YWHAZ-dependent mechanisms, with partial involvement of PI3K-Akt signaling and additional pathways likely contributing. The dissociation between phenotypic rescue and Akt phosphorylation restoration suggests that YWHAZ may promote invasion through both PI3K-Akt-dependent and PI3K-Akt-independent mechanisms. These findings, obtained in ACHN cells, suggest that the FN1/YWHAZ axis may serve as a candidate prognostic marker and a potential target for future preclinical investigation in RCC, though validation in additional RCC/ccRCC models is warranted.

## Data Availability

The raw sequencing data have been submitted to the NCBI Sequence Read Archive (SRA) under BioProject accession PRJNA1423128. Accession numbers for individual samples can be found in the associated metadata.
