# Supplementary material for: FN1 promotes invasion of RCC via YWHAZ-associated signaling with partial involvement of the PI3K-Akt pathway
Source: Front Oncol. 2026 Oct 14;16:1974418. doi: 10.3389/fonc.2026.1974418 (PMC13713405; doi:10.3389/fonc.2026.1974418)
Supplement: Supplementary file 1 [file SupplementaryFile1.docx]

**Supplementary Figures**

**Supplementary Figure S1. FN1 knockdown inhibits cell proliferation but does not affect cell migration.**

A.CCK-8 assay assessing the impact of FN1 knockdown on ACHN cell proliferation. ****P＜0.0001

B.Wound healing assay evaluating the effect of FN1 knockdown on ACHN cell migration. ns, not significant.

**Supplementary Figure S2. DESeq2 mean-dispersion plot of gene expression.**

The black dots represent the gene-wise dispersion estimates (gene-est), the red line shows the fitted mean-dispersion trend (fitted), and the blue circles represent the final dispersions after empirical Bayes shrinkage (final).

**Supplementary Figure S3. The original images of blots exhibited in Figure 2B.**

**Supplementary Figure S4. The original images of blots exhibited in Figure 4E.**

**Supplementary Figure S5. The original images of blots exhibited in Figure 6B.**

**Supplementary Figure S6. The original images of blots exhibited in Figure 6D.**
